# Comparison of the clinical outcomes between conventional intracytoplasmic sperm injection (ICSI) and PIEZO-ICSI in women undergoing the first cycle of in-vitro fertilization

**DOI:** 10.1371/journal.pone.0330951

**Published:** 2025-08-29

**Authors:** Chun Kyu Lim, Sun Hee Shin, Go-woon Kim, Hwa Yeong Kim, Hye Jin Cha, Jung Jae Ko, Kyung-Ah Lee, Hyejin Ryu, Kyung Eun Lee, Seyul Han, Wonyun Choi

**Affiliations:** 1 CHA University Fertility Center Ilsan, CHA University Ilsan Medical Center, Goyang-si, Gyeonggi-do, Republic of Korea; 2 CHA University Global IVF Group, Seongnam-si, Gyeonggi-do, Republic of Korea; Friedrich-Loeffler-Institute, GERMANY

## Abstract

The effectiveness of PIEZO-ICSI (P-ICSI) compared to conventional ICSI (C-ICSI) is still controversial. The only confirmed effectiveness of P-ICSI compared to C-ICSI is that it can reduce the number of degenerated oocytes after ICSI. This study included 100 patients undergoing their first IVF cycle. The patients were randomly assigned to C-ICSI group and P-ICSI group, 50 patients per each group. A total of 2,434 oocytes were retrieved from 100 patients. Among them, 1,527 oocytes with visible meiotic spindle were inseminated by C-ICSI or P-ICSI (778 by C-ICSI, 749 by P-ICSI). Fertilization, degeneration after ICSI and blastocyst development were compared between the C-ICSI group and P-ICSI group. Among the embryos developed to blastocysts, good quality blastocysts were vitrified. The frozen-thawed embryo transfer (FET) cycles were carried out in 42 patients of C-ICSI group and in 45 patients of P-ICSI group. Clinical pregnancy outcomes were analyzed. The differences between C-ICSI group and P-ICSI group were statistically analyzed using Mann-Whitney U test or chi-Square test. Patient age was not different between C-ICSI group and P-ICSI group (33.5 ± 2.7 vs. 32.9 ± 2.4, P = 0.12114). Normal fertilization rate of P-ICSI group (584/749, 78.0%) was significantly higher than that of C-ICSI group (565/778, 72.6%, P = 0.0176). The oocyte degeneration rate after ICSI was significantly higher in C-ICSI group (49/778, 6.3%) than in P-ICSI group (24/749, 3.2%, P = 0.0055). Among the fertilized oocytes, 21 did not cleave; 14 were from C-ICSI group (2.48%) and 7 from P-ICSI group (1.20%, P = 0.1250). Blastocyst formation rate (65.5% vs. 67.8%, P = 0.4485) and the rates of blastocyst that were available for vitrification (59.3% vs. 62.6%, P = 0.2724) were not different between C-ICSI group and P-ICSI group. Blastocyst formation rate on day 5 was significantly higher in P-ICSI group (50.3%) than in C-ICSI group (43.9%, P = 0.0367). In FET cycles, average age of patients was 33.6 ± 2.6 years old in C-ICSI group and that was 32.9 ± 2.3 years old in P-ICSI group. The average number of transferred embryos was 1.5 ± 0.5 in C-ICSI group and 1.2 ± 0.4 in P-ICSI group. The clinical pregnancy rate and the abortion rate of C-ICSI group were 64.3% and 18.5%, respectively. Those of P-ICSI group were 66.7% and 23.3%, respectively. This study showed that normal fertilization rate, the survival of oocytes after ICSI and blastocyst formation rate on day 5 were significantly higher in P-ICSI group than in C-ICSI group. Developmental arrest of zygotes was lower in P-ICSI group than in C-ICSI group. The number of embryos available for vitrification can be increased by implementing P-ICSI. P-ICSI has the potential to improve the development of zygotes into blastocysts.

## Introduction

After the introduction of intracytoplasmic sperm injection (ICSI) into human assisted reproduction technology (ART), ICSI has become a cornerstone in the management of male-factor infertility [1]. The application of ICSI has been expanded from male factor infertility to non-male factor infertility and ICSI is currently the most prioritized insemination technique of the human in-vitro fertilization (IVF) cycles performed worldwide [2–6]. Advancements in ART has led to the development of alternative ICSI techniques, such as physiologic ICSI (PICSI), intracytoplasmic morphologically selected sperm injection (IMSI) and ICSI with polarized light microscopy [7–12]. All of these ICSI techniques essentially require the ooplasm to be aspirated into micro-injection pipette in order to rupture oolema. Oocytes are exposed to potential stress caused by physical force and dislocation of spindle during the procedures of conventional ICSI [13–15]. This aspiration procedure is very invasive and can damage oocyte after ICSI. Approximately 5 ~ 10% of oocytes undergoing ICSI are at risk of being damaged after ICSI [16]. The fertilization and development of survived oocytes after ICSI may be affected by the aspiration of ooplasm [13,17]. As an alternative technique to conventional ICSI, PIEZO-ICSI has been introduced into human assisted reproduction to reduce the damage of oocytes due to the aspiration of ooplasm in the late 1990s [18–20]. Prior to its introduction into human assisted reproduction, PIEZO-ICSI was first developed in mouse to decrease the degeneration of oocytes after conventional ICSI and to enhance fertilization of oocytes. In mice, the low fertilization rate (40 ~ 50%) after the implementation of conventional ICSI was improved to over 90% through the implementation of PIEZO-ICSI [21]. And then, its implementation had been extended to other animal species, such as rabbits, cats, horses and cattle. In those animal species, enhanced fertilization, successful production of good quality embryos and livebirth have been reported [22–24]. After its successful application into animal species, PIEZO-ICSI was first applied into human assisted reproduction, with clinical outcomes comparable to those of conventional ICSI [20].

Despite the potential advantage of reducing the damage of oocytes after ICSI, PIEZO-ICSI is currently implemented in a very limited number of infertility clinics, especially in Japanese clinics [18–20,25,26]. The implementation of PIEZO-ICSI doesn’t require the aspiration of ooplasm since it utilizes the high-speed movement of a blunt-ended injection pipette to penetrate zona pellucida and rupture oolemma. PIEZO-ICSI alleviate the potential stress that oocytes are exposed to during the procedure of ICSI. Therefore, PIEZO-ICSI is considered a less invasive ICSI than conventional ICSI. Studies on PIEZO-ICSI are very limited and the effectiveness of PIEZO-ICSI is still controversial due the limited implementation of PIEZO-ICSI. Most studies on the clinical outcomes of PIEZO-ICSI in assisted human reproduction have reported the improved fertilization and decreased degeneration after ICSI compared to conventional ICSI [18,19,25–29]. Several studies have shown that the number of embryos available for embryo transfer or cryopreservation can be increased in patients whose oocytes were inseminated by PIEZO-ICSI [27,28]. However, most of these studies have reported no differences in development into blastocyst and pregnancy outcomes between conventional ICSI and PIEZO-ICSI. More recent study has shown that PIEZO-ICSI might be beneficial for patients who had a poor prognosis in previous conventional ICSI cycles [27]. The study showed that clinical pregnancy rate and live birth rate as well as fertilization rate and survival rate increased after implementation of PIEZO-ICSI in those patients. Especially, PIEZO-ICSI was the most beneficial in patients whose fertilization rate was less than 50% or utilization rate was less than 20% in their previous conventional ICSI cycles. Compared to previous conventional ICSI cycles, the number of embryos utilized per cycle increased in PIEZO-ICSI cycle. However, the benefits of PIEZO-ICSI was not different between younger and older patients. Although the implementation of PIEZO-ICSI and studies on clinical outcomes on PIEZO-ICSI are very limited, the application of PIEZO-ICSI is currently expanding. Automation of the assisted reproductive techniques is one of areas of active research in the field of reproductive medicine. However, to date, very few steps of IVF process have been automated. While automated ICSI (ICSIA) has been studied, it is not yet applied clinically [30–32]. However, the birth of babies from embryos fertilized with ICSIA has been recently reported [33]. A prototype ICSIA robot was developed and PIEZO-ICSI was used in the ICSIA robot with artificial intelligence. Not all ICSI steps were not automated in the ICSIA robot but it was the first clinical application of ICSIA. PIEZO-ICSI has high usability but it has not been widely implemented to date, perhaps due to the inconvenience of the procedure. Compared to conventional ICSI, PIEZO-ICSI requires more time, more effort and extra equipment to implement. Furthermore, the equipment cannot be mounted on all types of micromanipulators. However, PIEZO-ICSI has many advantages. Therefore, if PIEZO-ICSI is well established clinically, it should be able to contribute to the improvement of clinical outcomes.

The purpose of this study was to assess whether PIEZO-ICSI could improve the clinical outcomes of ICSI compared to conventional ICSI. Recent studies showed that PIEZO-ICSI could improve clinical outcomes in patients who had a poor prognosis in previous cycles and be beneficial especially in women ≥38 years [27,28]. However, older patients were excluded in this study because a small number of oocytes were retrieved from them. This study was conducted in patients aged 37 years or younger undergoing their first cycle of IVF to exclude the effect of patients’ age and prognosis of previous cycles on ICSI outcomes. More studies that prove the effectiveness of PIEZO-ICSI are needed for PIEZO-ICSI to be widely implemented.

## Materials and methods

### Patients

This study was approved by CHA University Ilsan CHA Hospital Institutional Review Board (approval number: ICHA-2024-01-003). This is a retrospective study conducted from March 2024 to January 2025. Clinical outcomes after implementing a routine course for human in vitro fertilization and embryo transfer (IVF & ET) were analyzed in this study. Clinical outcomes were analyzed after anonymizing all information that could identify individual patients.

This study included a total of 100 patients under the age of 37 years, with normal body mass index (BMI), day 3 serum FSH < 10 IU/L and serum AMH > 1.0ng/ml. The patients underwent their first cycle of IVF. The patients were selected among patients undergoing ICSI due to male factor infertility (teratozoospermia). The patients were randomly allocated into conventional ICSI and PIEZO-ICSI, 50 patients per each group.

### Controlled ovarian stimulation and oocyte retrieval

To retrieve multiple oocytes, controlled ovarian stimulation was conducted using a gonadotropin-releasing hormone (GnRH) antagonist protocol as previously described [34]. Briefly, 150–300 IU of recombinant FSH (Puregon; MSD, Sydney, Australia or Gonal-F; Merck Serono, Sydney, Australia) was administered into patient daily starting on day 2 or 3 of the menstrual cycle. The GnRH antagonist (Cetrotide; Merck Serono, Sydney, Australia or Orgalutran; MSD, Sydney, Australia) was administered when two or more follicles reached a size of > 14 mm. In order to trigger, 5,000 or 10,000 IU of human chorionic gonadotropin (hCG, Ovidrel; Merck Serono, Sydney) was administered when two or more follicles reached a size of ≥ 18 mm. Oocyte-cumulus complexes (COCs) were retrieved transvaginally under ultrasound guidance 36h after hCG administration. The COCs were washed several times and incubated in Fertilization medium (Genea Biomedx, Sydney, Australia) after retrieval. Cumulus cells were removed using 80 IU/ml hyaluronidase (HYASE, Vitrolife, Sweden, AB) 1h after oocyte retrieval. Metaphase II oocytes with the first polar body and a visible spindle were inseminated with a single sperm using either C-ICSI or P-ICSI 2h after the removal of cumulus cells. After ICSI, the oocytes were washed several times and cultured in Fertilization medium.

### Conventional ICSI

Conventional ICSI was carried out under the LEIKA DMi8 inverted microscope (Wetzlar, Germany) equipped with Eppendorf TrasferMan 4r micromanipulator (Hamburg, Germany) using commercially available microinjection pipettes and holding pipettes. Sperm was immobilized by touching tail with injection pipette and aspirated into pipette tail first. Spindle of oocyte was observed using Oosight Meta imaging system (Hamilton Thorne Inc. Massachusetts, USA) by rotating the oocyte with injection pipette. Spindle was positioned at 12 or 6 o’clock and oocyte was held by holing pipette. Zona pellucida was penetrated at 3 o’clock and ooplasm was aspirated into injection pipette until the oolemma was ruptured. The aspiration of ooplasm was immediately stopped as soon as the oolemma was ruptured and sperm was released into ooplasm with the aspirated ooplasm. After ICSI, oocytes were washed several times and transferred to Fertilization medium.

### PIEZO-ICSI

PIEZO-ICSI was performed under the LEIKA DMi8 inverted microscope (Wetzlar, Germany) equipped with Eppendorf TrasferMan NK2 microinjectors using ultrathin walled PIEZO-ICSI pipettes (PINU06–25FT; Prime Tech Ltd.) and holding pipettes. The operation liquid was put in the middle of the injection pipette. The operation liquid was pushed to the end of the pipette and 6% PVP solution was aspirated into the pipette using pneumatic injector. Sperm was immobilized by touching tail with injection pipette and aspirated into injection pipette tail first. Spindle of oocyte was observed using Oosight Meta imaging system (Hamilton Thorne Inc. Massachuestts, USA). Spindle was positioned at 12 or 6 o’clock and oocyte was held by holing pipette. Zona pellucida (ZP) was penetrated at 3 o’clock using piezo pulses (intensity 5, speed5). After injection pipette was withdrawn out of ZP, sperm was pushed to the end of the pipette, expelling the remnant ZP. The pipette was inserted into the oocyte and oolemma was ruptured using a single piezo pulse (intensity 3, speed 1). Sperm was released into the ooplasm and pipette was pulled out without any aspiration of ooplasm. After ICSI, oocytes were washed several times and transferred to Fertilization medium.

### Embryo culture

Oocytes were transferred into droplet of Fertilization medium covered with mineral oil after ICSI. Fertilization was assessed 16 ~ 18 hours after ICSI. Normal fertilization was determined when two clear pronuclei and second polar body were observed. Abnormal fertilization was determined when one pronucleus or three or more pronuclei were observed. Oocytes were classified as degeneration when the oocytes were lysed immediately after ICSI or at the time of fertilization assessment. Fertilized oocytes were transferred into Cleavage medium (Genea Biomedx, Sydney, Australia) after fertilization assessment and cultured individually under 5% O_2_, 6% CO_2_ and 37°C. Embryos were transferred to Blastocyst medium (Genea Biomedx, Sydney, Australia) at 48 hours after culture in Cleavage medium and cultured for 48 ~ 72 hours. Embryonic development and blastocyst formation were assessed at 48 hours after culture in Blastocyst medium. Good quality blastocysts available for vitrification were selected and vitrified. At 72 hours of culture in Blastocyst medium, embryonic development and blastocyst formation were assessed and good quality blastocysts were vitrified. All good quality blastocysts were vitrified in all cycles and frozen-thawed embryo transfer cycles were performed.

### Vitrification, warming and embryo transfer

Before vitrifying blastocysts, a single laser pulse was applied to the trophectoderm at the cellular junction among cells to induce collapsing of blastocoel. The artificial shrinkage was carried out using RI Saturn Laser System (CooperSurgical, Ballerup, Denmark) on the warm stage heated to 37°C. Blastocysts were vitrified one by one. Vitrification and warming of blastocysts were performed using vitrificaiton and warming solutions that were prepared in the laboratory using Quinn’s Advantage ^TM^ Medium with HEPES (SAGE, Trumbull, USA), Human Serum Albumin (HAS; SAGE, Trumbull, USA) and cryoprotectants including dimethyl sulfoxide (DMSO; D2650, Sigma-Aldrich, St. Louis, USA), ethylene glycol (102466, Sigma-Aldrich, St. Louis, USA) and sucrose (S1888, Sigma-Aldrich, St. Louis, USA). Blastocyst was transferred to HEPES-buffered culture medium supplemented with 20% HSA and incubated for 30 s. Then the blastocyst was transferred to equilibration solution containing 10% ethylene glycol and 10% DMSO and incubated for 2 min 30 sec. The blastocyst was transferred to vitrification solution containing 20% ethylene glycol, 20% DMSO and 0.3 mol/L sucrose for 20 s. One blastocyst was loaded on electron microscope gold grid (Plain Grid with Handle, 400 mesh, Hole 38μm; Graticules Optics, Cambridge, UK). The blastocyst was plunged into liquid nitrogen and stored until embryo transfer.

The afternoon before embryo transfer in frozen-thawed embryo transfer cycle, one or two blastocysts were warmed. For warming, vitrified blastocyst was placed into HEPES-buffered culture medium supplemented with 20% HSA containing 0.6 mol/L sucrose for 3 min. Then, the blastocyst was sequentially incubated in 0.5 mol/L sucrose for 2 min 30 sec and 0.25 mol/L sucrose for 2 min 30 sec. Lastly, the blastocyst was incubated in HEPES-buffered culture medium supplemented with 20% HSA for 1 min. After warming, the blastocyst was transferred into Blastocyst medium (Genea Biomedx) and cultured under 5% O_2_, 6% CO_2_ and 37°C until embryo transfer.

One or two blastocysts were transferred into the uterus based on the patient’s age and the blastocyst quality. Single blastocyst was transferred in patients under 34 years of age and two blastocysts were transferred in patients over 35 years of age. In patients over 35 years of age, single blastocyst was transferred when the patients requested single blastocyst to be transferred. Blastocysts were incubated in EmbryoGlue (Vitrolife, Sweden, AB) for 10 minutes prior to transfer. The time that the blastocysts were incubated in EmbryoGlue did not exceed 20 minutes. Clinical pregnancy was confirmed after gestational sac or fetal heartbeat was identified using ultrasound sonography. Miscarriage was determined when fetus was lost spontaneously before the first trimester of gestation.

### Statistical analysis

The results were expressed as percentages or the mean ± standard deviation. Statistical analyses were carried out using the Statistics Package for Social Sciences (SPSS Inc., Chicago, IL, USA). Fisher’s exact test was used for the analyses of categorical data (fertilization, development, utilization and pregnancy results). Mann Whitney U-test was used for the analyses of continuous data. *P* values <0.05 were considered to be statistically significant.

## Results

### Patients

A total of 100 patients was included in this study. The main indication of these patients was male factor infertility. Normal morphology of male partners’ sperm evaluated according to Tygerberg’s strict criteria [35] was less than 2%. Semen volume, sperm concentration, sperm motility and morphology were not different between C-ICSI group and P-ICSI group. C-ICSI or P-ICSI was implemented in 50 patients per each group. There were no significant differences in maternal age, AMH concentration, day 3 serum FSH concentration between C-ICSI group and P-ICSI group. BMI was significantly higher in patients undergoing P-ICSI (23.4 ± 3.7) than in those undergoing C-ICSI (22.0 ± 3.5, P = 0.02926) (Table 1).

**Table 1:** Demographic characteristics of the patients.

	Conventional ICSI	PIEZO-ICSI	P-value
No. of patients	50	50	
No. of cycles	50	50	
Maternal Age	33.5 ± 2.7	32.9 ± 2.4	0.12114
Female BMI	22.0 ± 3.5	23.4 ± 3.7	0.02926
AMH (ng/ml)	4.9 ± 2.9	4.1 ± 2.2	0.33706
FSH (IU/L)	7.0 ± 2.3	7.2 ± 1.9	0.70394
Semen volume (ml)	3.0 ± 1.2	3.1 ± 1.4	0.88076
Sperm count (X10^6^/ml)	83.3 ± 40.5	77.3 ± 44.9	0.34722
Motile sperm count (X10^6^/ml)	39.0 ± 23.1	37.0 ± 23.7	0.70394
Sperm motility (%)	45.5 ± 11.5	47.4 ± 10.2	0.70394
Sperm morphology (%)	1.1 ± 0.4	1.3 ± 0.8	0.9442

### Fertilization and embryonic development

A total of 2,434 oocyte-cumulus complexes were retrieved from 100 patients, 1,245 oocytes from C-ICSI group and 1,189 oocytes from P-ICSI group. Among them, 1,527 metaphase II oocytes with visible spindle were injected with sperm by C-ICSI or P-ICSI. Seven hundred seventy-eight oocytes were inseminated by C-ICSI and 749 oocytes by P-ICSI. There were no significant differences in number of retrieved oocytes (24.9 ± 8.0 vs 23.8 ± 7.8), number of injected oocytes (15.6 ± 6.7 vs 15.0 ± 5.1) and rates of metaphase II oocytes (62.5% vs 63.0%) between C-ICSI group and P-ICSI group (Table 2). Five hundred sixty-five oocytes (72.6%) were normally fertilized by C-ICSI and 584 oocytes (78.0%) by P-ICSI, respectively. Normal fertilization rate of P-ICSI group was significantly higher than that of C-ICSI group (P = 0.0176). After ICSI, 49 oocytes (6.3%) were degenerated in C-ICSI group and 24 oocytes (3.2%) in P-ICSI group. Oocyte degeneration rate of C-ICSI group was significantly higher than that of P-ICSI group (P = 0.0055).

**Table 2:** Comparison of ICSI outcomes between conventional ICSI and PIEZO-ICSI.

No. of	Conventional ICSI	PIEZO-ICSI	P-value
Retrieved oocytes (mean±SD)	1,245 (24.9 ± 8.0)	1,189 (23.8 ± 7.8)	0.5157
Injected oocytes (mean±SD)	778 (15.6 ± 6.7)	749 (15.0 ± 5.1)	0.88866
Normally fertilized oocytes (2PN)	565 (72.6%^a^)	584 (78.0%^a^)	0.0176
Abnormally fertilized oocytes (1PN, 3PN)	26 (3.3%^a^)	17 (2.3%^a^)	0.2191
Degenerated oocytes	49 (6.3%^a^)	24 (3.2%^a^)	0.0055

^a^percent per injected oocytes.

Among normally fertilized oocytes, 21 zygotes did not cleave after pronuclei disappeared. Fourteen zygotes did not cleave in C-ICSI group (2.48%) and seven in P-ICSI group (1.20%). The rate of zygotes that did not cleave was higher in C-ICSI group than in P-ICSI group but there was no significant difference between them (P = 0.1250). Seven hundred fifty-two embryos of 1,128 embryos that cleaved after pronuclei disappeared developed to blastocysts. Three hundred sixty-one embryos developed to blastocysts in C-ICSI group (361/554, 65.2%) and 391 embryos developed to blastocysts in P-ICSI group (391/581, 67.3%). More embryos developed to blastocysts in P-ICSI group (7.8 ± 3.8) than in C-ICSI group (7.2 ± 3.5) but there was no significant difference between them (P = 0.53526). Three hundred twenty-seven blastocysts were vitrified in C-ICSI group (327/554, 59.0%) and 361 blastocysts in P-ICSI group (361/581, 62.1%). Blastocyst formation rate and usable blastocysts rate were higher in P-ICSI group than in C-ICSI group but there were no significant differences between C-ICSI group and P-ICSI group. Two hundred forty-two embryos developed to blastocysts (43.7%) on day 5 in C-ICSI group and 290 embryos developed to blastocysts in P-ICSI group (49.9%). Blastocyst formation rate on day 5 was significantly higher in P-ICSI group than in C-ICSI group (P = 0.0374) (Table 3).

**Table 3:** Comparison of embryonic development after ICSI between conventional ICSI and PIEZO-ICSI.

No. of	Conventional ICSI	PIEZO-ICSI	P-value
Embryos (mean±SD)	551 (11.0 ± 4.2)	577 (11.5 ± 4.3)	0.4965
Arrested embryos (%)^a^	14 (2.48%)	7 (1.20%)	0.1250
Blastocysts (mean±SD)	361 (7.2 ± 3.5)	391 (7.8 ± 3.8)	0.53526
% of blastocysts	65.5%	67.8%	0.4485
Blastocysts on day 5 (mean±SD)	241 (4.8 ± 3.1)	289 (5.8 ± 3.2)	
% of blastocysts on day 5/embryos	43.7%	50.1%	0.0367
% of blastocysts on day 5/blastocysts	66.8%	73.9%	0.0374
Vitrified blastocysts (mean±SD)	327 (6.5 ± 3.3)	361 (7.2 ± 3.4)	0.37346
% of vitrified blastocyst	59.3%	62.6%	0.2724

^a^number of embryos that did not cleave after pronuclei disappeared.

### Embryo transfer and pregnancy outcome

Frozen-thawed embryo transfer cycles were performed in 87 patients, 42 patients in C-ICSI group and 45 patients in P-ICSI group, respectively. The average maternal age was 33.6 ± 2.6 years old in C-ICSI group and 32.9 ± 2.3 years old in P-ICSI group. Sixty-three blastocysts (1.5 ± 0.5) were transferred in C-ICSI group and 52 blastocysts (1.2 ± 0.4) were transferred in P-ICSI group. Clinical pregnancies were confirmed in 57 patients, 27 patients in C-ICSI group (64.3%) and 30 patients in P-ICSI group (66.7%). Ten patients delivered babies and seven pregnancies are on-going in C-ICSI group. Five pregnancies were aborted within the first trimester of gestation (18.5%) and five patients received pregnancy care at other hospital due to the distance from hospital. Among clinical pregnancies, 24 pregnancies were singleton, two were twin and one was triplet pregnancy. In the triplet pregnancy, two blastocysts were transferred and the triplet pregnancy was resulted in. A total of 31 gestational sacs were confirmed. In case where two blastocysts were transferred and triplet pregnancy was resulted in, it was analyzed that two blastocysts were implanted. Therefore, the implantation rate of C-ICSI group was 47.6%. Eleven patients delivered babies and 11 pregnancies are on-going in P-ICSI group. Seven pregnancies were aborted within the first trimester of gestation (23.3%) and one patient received pregnancy care at other hospital. All pregnancies were singleton pregnancies. Thirty gestational sacs were confirmed and the implantation rate was 57.7%. (Table 4).

**Table 4:** Comparison of pregnancy outcomes between conventional ICSI and PIEZO-ICSI.

	Conventional ICSI	PIEZO-ICSI	TOTAL
No. of frozen-thawed embryo transfer cycles	42	45	87
Maternal age	33.6 ± 2.6	32.9 ± 2.3	33.2 ± 2.5
No. of transferred embryos (mean±SD)	63 (1.5 ± 0.5)	56 (1.2 ± 0.4)	119 (1.4 ± 0.5)
No. of clinical pregnancies (%^a^)	27 (64.3)	30 (66.7)	57 (65.5)
Deliveries (%^b^)	10 (37.0)	11 (36.7)	21 (36.8)
On-going pregnancies (%^b^)	7 (25.9)	11 (36.7)	18 (31.6)
Singleton pregnancies	24	30	54
Twin pregnancies	2		2
Triplet pregnancies	1		1
Abortions (%^b^)	5 (18.5)	7 (23.3)	12 (21.1)
Lost follow-ups (%^b^)	5 (18.5)	1 (3.3)	6 (10.5)
No. of G-sac (implantation rate, %^c^)	31 (47.6)	30 (57.7)	61 (51.3)

^a^percent per frozen-thawed embryo transfer cycle.

^b^percent per clinical pregnancy.

^c^percent per transferred embryos; In conventional ICSI group, the implantation rate was analyzed that two blastocysts were implanted since triplet pregnancy was resulted from transfer of two blastocysts.

## Discussion

This study showed that PIEZO-ICSI could significantly increase fertilization rate and decrease degeneration of oocytes after ICSI in patients undergoing their first IVF cycle. The improvement of fertilization and survival of oocytes after PIEZO-ICSI were consistent with previous studies [18–20,25,27–29]. In most of those studies, PIEZO-ICSI could improve the clinical outcomes in patients who had poor clinical outcomes in their previous cycles [19,25,27,28]. Unlike recent studies, this study compared the outcomes of conventional ICSI and PIEZO-ICSI in patients undergoing their first cycle of IVF and showed that the improved ICSI outcomes could be achieved by PIEZO-ICS in those patients. The patients aged 37 years or younger and with more than 10 retrieved oocytes were included in this study to exclude the patients with a poor prognosis. The AMH level, FSH level and BMI of the patients were within the normal range. This study suggested that PIEZO-ICSI may improve the ICSI outcomes not only in the patients with a poor prognosis but also in the patients with a good prognosis. Based on the studies to date, including this study, it is suggested that PIEZO-ICSI may not only be a good alternative to conventional ICSI but may also show clinical outcomes comparable to or better than conventional ICSI.

Most studies reporting the outcomes of PIEZO-ICSI have focused on the degeneration and fertilization of oocytes after ICSI. Therefore, embryonic development after PIEZO-ICSI was not well documented in literatures. Conflicting results have been reported regarding the cleavage rate. Yanagida *et al*. have reported that cleavage rate was not different between conventional ICSI and PIEZO-ICSI [19]. Takeuchi *et al*. have shown that cleavage rate was significantly higher in PIEZO-ICSI than in conventional ICSI [18]. Fujii *et al*. have reported that cleavage rate was higher in conventional ICSI than in PIEZO-ICSI but there is no statistical difference [25]. In this study, cleavage rate was higher in PIEZO-ICSI group than in conventional ICSI group but there is no statistical difference. These different results in each study might be due to the different patient populations in respective study as well as the lack of studies. In contrast to the cleavage rate, the blastocyst formation rate has shown consistent results. The blastocyst formation rate and the utilization rate were not different between conventional ICSI group and PIEZO-ICSI group but the number of blastocyst and the number of utilized embryos were higher in PIEZO-ICSI group than in conventional ICSI group [17,25,27–29]. Consistent with recent studies, the blastocyst formation rate and the utilization rate were comparable between conventional ICSI group and PIEZO-ICSI group in this study. And the number of blastocysts and the number of utilized embryos were higher in PIEZO-ICSI group than in conventional ICSI group. But the numbers were not statistically significant between conventional ICSI group and PIEZO-ICSI group. Although blastocyst formation rate was not different between conventional ICSI group and PIEZO-ICSI group, the blastocyst formation rate on day 5 was significantly higher in PIEZO-ICSI group than in conventional ICSI group. Fujii et al. reported that blastocyst formation rate was not different on day 5 after oocyte retrieval between conventional ICSI and PIEZO-ICSI [25]. Except for the study by Fujii et al., no other studies have investigated the blastocyst formation rate depending on the day after oocyte retrieval. This different result between this study and Fujii et al. may be caused by different study population or different PIEZO-ICSI method. Sperm was immobilized by touching the tail of sperm with injection pipette in this study but continuous PIEZO pulses were driven to immobilize sperm in Fujji et al. The sperm immobilized by touching and the sperm immobilized by PIEZO pulses showed different characteristics [19]. Sperms immobilized with PIEZO pulses showed rapider membrane permeability to membrane-impermeable stain and rapider induction of Ca^2^+ oscillation than sperms immobilized by touching. The differences of immobilized sperms may result in the differences of embryonic development as well as oocyte fertilization.

In this study, all good quality blastocysts were vitrified and frozen-thawed blastocysts were transferred. Clinical pregnancy rate was not different between conventional ICSI group and PIEZO-ICSI group. However, the number of transferred blastocysts was smaller in PIEZO-ICSI group than in conventional ICSI group. Improved pregnancy rates have been reported when PIEZO-ICSI was performed in patients who had a poor prognosis in the previous cycle [19,27]. According to Caddy *et al*., pregnancy rate was improved when fresh embryos, especially blastocysts, were transferred. Pregnancy rate was not improved when cleavage stage embryos were transferred. And pregnancy rate was not different between conventional ICSI group and PIEZO-ICSI group when frozen-thawed blastocysts were transferred. Consistent with the study, pregnancy rate was not different between conventional ICSI group and PIEZO-ICSI group in this study. In our center, fresh embryos are rarely transferred. All good quality embryos are frozen and frozen-thawed embryos are transferred in most human IVF-ET cycles. Therefore, we could not analyze the pregnancy outcomes of fresh embryo transfer cycles in conventional ICSI group or PIEZO-ICSI group. Typically, fresh blastocyst transfer is performed on day 5 after oocyte retrieval. In this study, the number of blastocyst was not different between conventional ICSI group and PIEZO-ICSI group. However, the number of blastocyst formed on day 5 was higher in PIEZO-ICSI group than conventional ICSI group. Therefore, if the fresh blastocyst transfer had been performed on day 5, the possibility of selecting good quality blastocysts would have been higher in PIEZO-ICSI group than in conventional ICSI group, and pregnancy rate might have been different between conventional ICSI group and PIEZO-ICSI group. To date, embryonic development and pregnancy outcomes after PIEZO-ICSI have not been well studied. In this study, the number of patients in each group was too small to compare pregnancy outcomes such as clinical pregnancy rate, abortion rate and implantation rate between conventional ICSI group and PIEZO-ICSI group. Studies involving larger number of patients are needed to show which group is superior to the other. This study is a retrospective study based on 50 patients in each group. If this had been conducted as a prospective study with all relevant factors well controlled, different results might have been obtained. These are the limitations of this study. Further large-scale randomized control studies should be conducted to verify the effectiveness of PIEZO-ICSI.

In conclusion, this study has shown that, compared to conventional ICSI, PIEZO-ICSI could improve fertilization and survival of oocytes after ICSI. Blastocyst formation rate on day 5 was higher in PIEZO-ICSI group than in conventional ICSI group. Overall, PIEZO-ICSI outcomes, except for the outcomes above, were better than those of conventional ICSI but there were no significant differences. These no significant differences between outcomes of them may be due to the patients of this study and frozen-thawed embryo transfer, rather than fresh embryo transfer. The implementation of PIEZO-ICSI could reduce the number of degenerated oocytes after ICSI in infertile patients undergoing their first cycle of IVF-ET. In general, many oocytes are retrieved in those patients. Since the number of degenerated oocytes could be reduced in the patients with high number of oocytes retrieved, the implementation of PIEZO-ICSI could also reduce the number of degenerated oocytes in patients with low number of oocytes retrieved. Just by being able to reduce the degeneration of oocytes after ICSI, PIEZO-ICSI might be beneficial for patients with a small number of retrieved oocytes. PIEZO-ICSI might be worth of trying in patients who had poor outcomes of conventional ICSI in previous cycles. Further studies to evaluate the effectiveness of PIEZO-ICSI in those patients are needed and are ongoing in our center.

## Supporting information

S1 Data Supporting information including raw data used for analyses. (XLSX)
